# Relationship between nutrient intakes in the transition phase and postnatal growth of preterm infants: a systematic review

**DOI:** 10.1186/s13052-022-01406-3

**Published:** 2023-01-21

**Authors:** Na Wang, Jia Zhang, Bo Wang, Zhangbin Yu, Jun Zhang, Linlin Qu, Bin Tang

**Affiliations:** 1 Department of Pediatric, the Affiliated Suqian First People’s Hospital of Nanjing Medical University, Jiangsu, China; 2 Department of Neonatology, The Second Clinical MedicalCollege, Shenzhen People’s HospitalJinan UniversityThe First Affiliated Hospital, Southern University of Science and Technology), Shenzhen, Guangdong China

**Keywords:** Parenteral nutrition, Enteral nutrition, Growth, Preterm infant, Systematic review

## Abstract

Nutrition practices for preterm infants include phases of parenteral nutrition, gradually interrupted parenteral nutrition (transition phase), and full enteral nutrition. However, nutrition management during the transition phase is frequently overlooked. This review examined the relationship between nutrient intake during the transition phase and preterm infant growth. PubMed, Embase, Web of Science, Cochrane, Chinese National Knowledge Infrastructure Database, Wanfang Database, and Chinese Science and Technique Journals Database were searched for studies examining the relationship between nutrient intake during the transition phase and postnatal growth of preterm infants from each database's earliest inception through February 28, 2022. The quality of the studies was assessed using the Newcastle–Ottawa scale. A total of three studies conducted in the USA, Italy and China met the inclusion criteria. The growth indicators were extrauterine growth restriction (weight < 10th percentile for post-menstrual age) or inadequate weight growth velocity (growth velocity < 15 g/kg/d) at discharge or the end of the transition phase. The transition phase was divided into two periods in two studies: the early period (parenteral energy intake > 50% of total energy intake) and the late period (enteral energy intake > 50% of the total energy intake). The cumulative protein intake in the transition phase was generally lower in preterm infants with extrauterine growth restriction or inadequate weight growth velocity, especially in the early transition phase. The deficiency of energy and protein intake during the transition phase cannot be explicitly determined due to differences in growth indicators and definitions of the transition phase. However, enteral protein intake should be closely monitored in the early transition phase to ensure a better growth rate for preterm infants. To elucidate potential associations, further well-designed research will be required.

## Introduction

In preterm infants born during the rapid fetal growth stage, the intrauterine growth rate is not reached after birth [1]. Parenteral and enteral nutritional support for newborns is based on continuous nutrition guidelines [2–4], and the risk of postnatal growth failure (PGF) in preterm infants remains high [5, 6]. Moreover, PGF is associated with subsequent poor neurodevelopment outcomes [7]. Therefore, the prevention of PGF in preterm infants remains a challenge for physicians.

Nutrient intakes during preterm infant hospitalization have long been thought to be an important factor affecting postnatal growth; however, implementing a standardized parenteral nutrition program for very low birth weight infants significantly improved postnatal growth [8]. According to a New York State perinatal quality collaborative report, enteral feeding practices vary, and extrauterine growth retardation (EUGR) is more prevalent in preterm infants [9]. To optimize the growth of preterm infants, Roggero et al. [10] suggested three phases of nutrition intervention: parenteral nutrition (PN), gradually interrupted parenteral nutrition (transition phase, TP) and complete enteral nutrition (EN). Previous studies on preterm infant nutrition tended to emphasize early or late parenteral and enteral nutrition [11, 12], while less attention was paid to the impact of nutrient intakes in the TP from PN to EN on preterm infants.

Miller et al. [13] proved that the poor growth of preterm infants in TP from PN to EN can predict the growth failure of preterm infants at discharge. Growth failure of preterm infants at discharge is not significantly related to the PN-only phase's poor growth. Since then, the concept of TP has attracted much attention [14–22].

Optimizing postnatal growth has always been the goal clinicians try to achieve [23, 24]. The relationship between nutrient intake in the TP and growth is undoubtedly a noteworthy highlight in the three nutrition phases that preterm infants undergo. The purpose of this review was to assess the relationship between nutrient intakes in the TP and preterm infant growth to help clinicians improve nutrition management in this setting.

## Materials and methods

A systematic review of the literature on the relationship between nutrient intakes in the TP and postnatal growth of preterm infants was conducted based on the Preferred Reporting Items for Systematic Reviews and Meta-Analyses (PRISMA) guidelines [25].

### Information sources and searches

Literature searches were performed on PubMed, Embase, Web of science, Cochrane, Chinese National Knowledge Infrastructure Database (CNKI), Wanfang Database, and Chinese Science and Technique Journal Database (VIP). From the earliest inception of each database through February 28, 2022, all articles in any language were eligible for inclusion. A combination of MeSH terms and other equivalent terms were applied together with truncated search terms and Boolean operators to achieve broader coverage, resulting in the search detail as follows: ("infant, low birth weight" OR "Low-Birth-Weight" OR "low birth weights" OR "infant, premature" OR "premature" OR "preterm") AND ("partial parenteral" OR "transition" OR "transfer" OR "transitional" OR "weaned" OR "weaning") AND ("nutrition" OR "nutritional" OR "energy" OR "protein" OR "amino acid").

### Inclusion and exclusion criteria

Original articles on nutrient intake during the TP from PN to EN and the growth of preterm infants / low birth weight infants were eligible for this review. The inclusion criteria were as follows: (1) population: preterm infants or infants of low birth weight. Preterm infants include very preterm infants or extremely preterm infants. Low birth weight infants include very low birth weight infants or extremely low birth weight infants; (2) exposure: nutrient intakes recommended by local nutrition guidelines; (3) outcome: the growth indicators should have been reported at the end of the TP or discharge such as weight percentage for post-menstrual age, weight z-score, or growth velocity; (4) the definitions of the TP and growth indicators were described. Exclusion criteria were as follows: (1) conference papers, animal experiments, reviews, and case reports; (2) incomplete data; (3) studies with the same participants but different purposes.

### Study selection and data extraction

Two independent researchers conducted the screening of the title and abstracts and the subsequent full-text assessment. Disagreements were resolved through discussion by the third researcher. Data extraction was performed separately by two researchers. Two researchers extracted the following data from included studies: 1) the authors' first name, publication year, study design, sample size, and study population; 2) description related to the TP (definition, start time, end time, and duration of the TP); 3) nutrient intakes in the TP (energy and protein); 4) growth indicators at the end of the TP and discharge (weight percentage for post-menstrual age and growth velocity). A third researcher verified the accuracy of the data extracted from each study.

### Quality assessment

The Newcastle–Ottawa scale (NOS) was used to evaluate the quality of case–control studies [26], which included eight items in three modules. Study population selection, comparability, and exposure. The scores on the scale ranged from one to nine. A score of seven or more stars indicated a good quality study; six stars indicated fair quality, and five or fewer stars indicated poor quality [27].

Data were grouped according to outcome indicators within exposure categories. The exposure in these categories was summarized. All the collected data were displayed in tables, where possible, and synthesized narratively. The lack of similarity between exposures and variations in outcome assessment and reporting precluded a meta-analysis.

## Results

### Study selection

**Fig.1:**
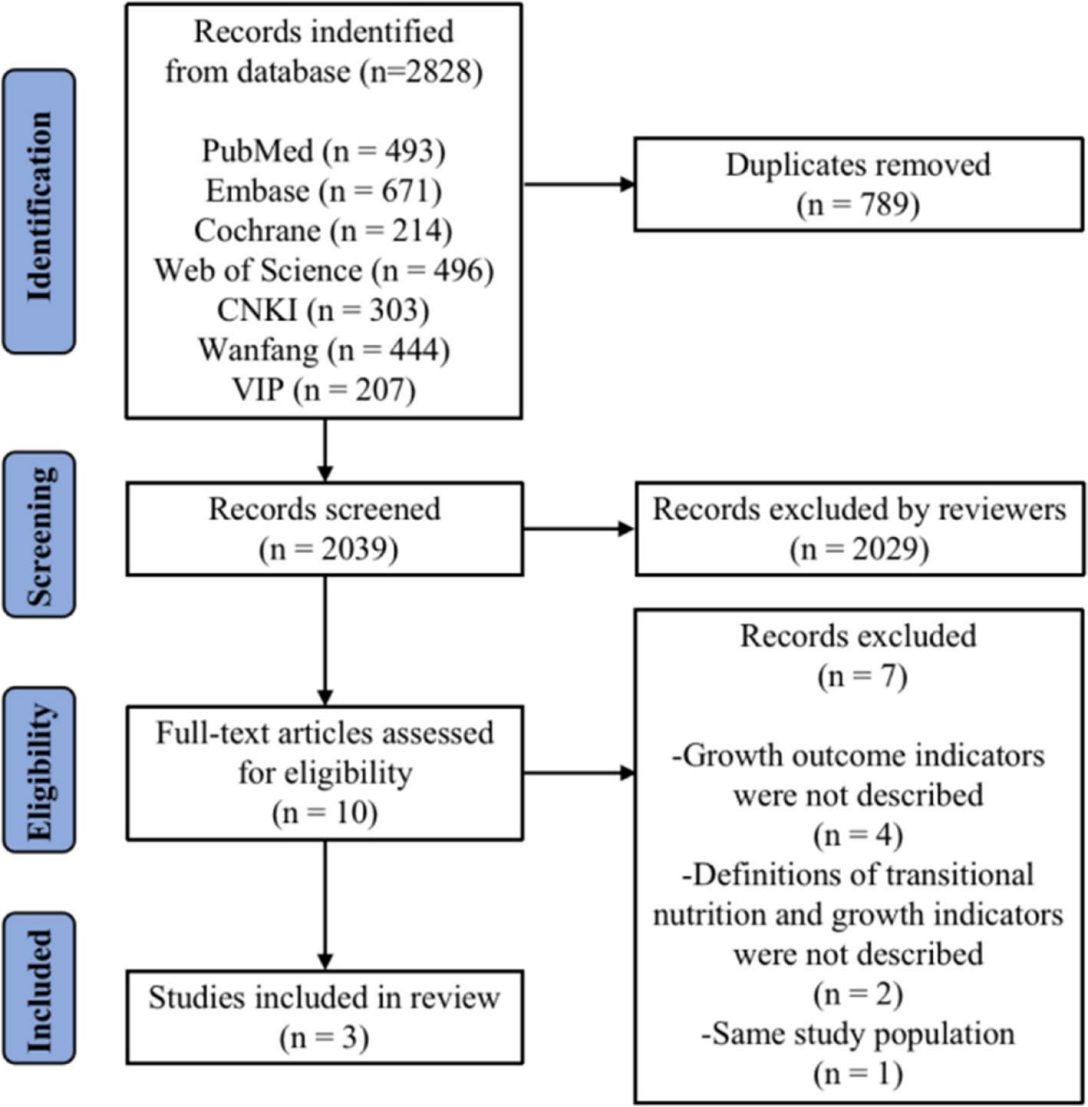
Flow diagram of the study selection process

A total of 2828 articles were obtained through preliminary screening. According to the inclusion and exclusion criteria, finally three articles were included in this systematic review [13, 19, 20]. The process of identification and selection is shown in Fig. 1.

### Description of included studies

The included studies were three case–control studies, all observational and single-center studies. One was conducted in the USA, one in Italy, and one in China. Two studies quantified the definition of TP according to the volume of enteral feeding [13.19], and the other study did not quantify the definition according to the corresponding volume of enteral feeding [20]. The weight percentiles for post-menstrual age were used as growth indicators in two studies. One study used growth velocity in the TP as an indicator of growth. The details of the study characteristics are provided in Table 1.

**Table 1:** Characteristics of the included studies

References	Year of publication	Design	N	Population	Definition of the TP	Nutritional guidelines	Growth indicators	Main Outcome
Miller et al. [13]	2014	Case–control	156	Gestational age < 32 w and birth weight < 2000 g	EN volume: 30 -160 ml/kg/d	Pediatric nutrition handbook of the American Academy of Pediatrics	Discharge weight < 10th percentile for post-menstrual age	1. Duration of the TP 2. Energy, protein and energy protein ratio in the TP 3. Weight percentile at discharge
Liotto et al. [19]	2020	Case–control	106	Birth weight < 1500 g	Start: the first day when PN began to decrease; End: the day when PN completely stopped, i.e. EN volume: 60—(150 ~ 180) ml/kg/d	European Society for Paediatric Gastroenterology Hepatology and Nutrition (ESPGHAN) guidelines	Growth velocity during the TP < 15 g/kg/d	Start and duration of the TP 2. Energy and protein in the TP 3. Growth velocity in the TP
Wang et al. [20]	2020	Case–control	40	Gestational age < 32 w and birth weight < 1500 g	Start: when AA infusion in PN increased to the goal of 3.5—4.0 g/kg/d; End: full intestinal feeding was reached	Chinese guideline for newborn nutrition support in neonates	Weight at the end of the TP < 10th percentile for post-menstrual age	1. Start and end time of the TP 2. AA in the TP 3. Weight percentile in the TP

*TP transition phase, AA amino acids, PN parenteral nutrition, EN enteral nutrition*

### Quality assessment

Based on the NOS quality assessment, two included studies were classified as moderate quality and one as high quality (Table 2).

**Table 2:** Newcastle–Ottawa Scale (case–control) for three studies included in this review

References	Selection				Comparability		Exposure			Results
	**Selection** **Definition Adequate**	**Selection** **Representativeness**	**Selection** **Control (community control)**	**Selection** **Definition of Control**	**Comparability** **Appropriate control**	**Comparability** **Other Factors**	**Ascertainment of exposure**	**Exposure Same Methodology**	**Non-Response Rate**	
Miller et.al. [13]	★	★	★	★	★	★	Not described	No	Not described	Fair
Liotto er.tal [19]	★	★	★	★	★	Not described	★	★	★	Good
Wang et.al. [20]	★	★	★	★	★	★	Not described	No	★	Fair

**★** Meet the scoring conditions

### Participant characteristics

A total of 302 participants were included in the studies, and study sample sizes ranged from 40 to 156. Although one of the included studies was carried out exclusively in preterm infants with birth weight < 1500 g, it can be concluded that their gestational age was less than 32 weeks according to the description of gestational age at birth [19]. Therefore, all included studies were carried out exclusively in preterm infants with gestational age < 32 weeks [13, 19, 20]. For all studies, participants’ birth weight Z-scores were reported ranging from -0.53 to 0.38 (mean, -0.51 ± 0.94) [19, 20], and birth weight percentile was 40% ± 17.8% [13]. After excluding small for gestational age (SGA) infants from the participating population, the incidence of EUGR in the TP was reported to range from 46 to 50% [13, 20], and the incidence of inadequate weight growth velocity (growth velocity < 15 g/kg/d) in the TP was 52.8% [19].

### Assessment of the TP

All studies divided the nutritional stages into the PN-only phase, TP, and EN phase, and the TP was all defined as the phase of gradual reduction of PN. A study defined TP quantitatively as the enteral feeding volume of 30—160 ml/kg/d [13]. Another study defined the TP quantitatively as the enteral feeding volume of 60—(150 ~ 180) ml/kg/d (Table 1) [19]. Two of the included studies further subdivided the TP into the early period dominated by PN (parenteral energy intake > 50% of the total energy intake, i.e., enteral feeding volume < 80 ml/kg/d) and the late period dominated by EN (enteral energy intake > 50% of the total energy intake, i.e., enteral feeding volume ≥ 80 ml/kg/d) [19, 20].

For all studies, the start time, duration, and end time of TP were 8.8 ± 6.3 days,14.7 ± 12.3 days, and 29.9 ± 9.8 days, respectively [13, 19, 20]. A study compared the start time and the end time of the TP between the EUGR group and the non-EUGR group, with no significant differences [5 (3, 8) vs. 4 (2, 9) days, 29 (15,58) vs. 28 (15,45) days, respectively] [20]. One study compared the start time and the duration of the TP between the inadequate weight growth velocity group and adequate weight growth velocity group, with no significant difference [16.3 ± 8.3 vs. 14.1 ± 8 days, 29 (15,58) vs. 21.3 ± 8.4 days, respectively] [19].

### Energy and protein intake in the TP

Protein and energy intake in the TP were assessed across three studies. One of the studies reported that protein intakes (< 3 g/kg/d), the nadir of cumulative protein (PN + EN), and the energy protein ratio (2.1 ± 0.5 g/kg and 2.3 ± 0.2 g/100 kcal, respectively) [13]. Another study reported that the cumulative energy and protein (PN + EN) in the TP (105.6 ± 15.9 kcal/kg/d and 3.4 ± 0.6 g/kg/d, respectively) [19]. The remaining study reported amino acid metabolic patterns in the TP [20].

Two studies dividing the TP into the early and late stages described protein (parenteral and enteral) and energy (parenteral and enteral) intake in the early and late stages [19, 20], as shown in Table 3.

**Table 3:** Comparison of nutrient intakes in the TP in very preterm infants with and without EUGR / inadequate weight growth velocity

References	N	Nutrient intakes in the TP		EUGR / inadequate weight growth velocity	Non-EUGR / adequate weight growth velocity	*P*
Liotto et al. [19]	56/50	In the early TP	Parenteral protein intake (g/kg/day)	2.36 ± 0.7	2.45 ± 0.8	0.53
			Parenteral energy intake (Kcal/kg/day)	59.4 ± 18.6	61.9 ± 24.1	0.54
			Enteral protein intake (g/kg/day)	1.09 ± 0.5	1.33 ± 0.7	0.05
			Enteral energy intake (kcal/kg/day)	45.5 ± 16.4	51.5 ± 21.3	0.10
			Cumulative energy in PN + EN (kcal/kg/d)	-	-	-
			Cumulative protein in PN + EN (g/kg/d)	-	-	-
		In the late TP	Parenteral protein intake (g/kg/day)	1.28 ± 0.7	1.74 ± 0.9	0.01
			Parenteral energy intake (Kcal/kg/day)	30.0 ± 17.0	43.6 ± 22.6	< 0.001
			Enteral protein intake (g/kg/day)	1.82 ± 0.8	1.73 ± 0.8	0.58
			Enteral energy intake (Kcal/kg/day)	72.4 ± 20.0	64.2 ± 26.5	0.07
			Cumulative energy in PN + EN (kcal/kg/d)	-	-	-
			Cumulative protein in PN + EN (g/kg/d)	-	-	-
Wang et al. [20]	20/20	In the early TP	Parenteral amino acid intake (g/kg/d)	2.55 ± 0.42	2.52 ± 0.41	0.78
			Parenteral energy intake (kcal/kg/d)	62.10 ± 7.98	62.06 ± 10.39	0.99
			Enteral amino acid intake (g/kg/d)	0.51 (0.23, 0.87)	0.75 (0.30, 1.61)	0.03
			Enteral energy intake (kcal/kg/d)	19.52 ± 7.24	27.16 ± 14.42	0.04
			Cumulative energy in PN + EN (kcal/kg/d)	81.62 ± 8.43	89.23 ± 9.01	0.01
			Cumulative amino acid in PN + EN (g/kg/d)	-	-	-
		In the late TP	Parenteral amino acid intake (g/kg/d)	1.41 (0.88, 2.05)	1.38 (1.03, 1.79)	0.63
			Parenteral energy intake (kcal/kg/d)	34.43 (21.44, 54.45)	32.90 (25.37, 46.01)	0.41
			Enteral amino acid intake (g/kg/d)	1.74 (0.82, 2.25)	1.80 (0.99, 2.23)	0.75
			Enteral energy intake (kcal/kg/d)	64.82 (28.60, 81.17)	65.38 (45.19, 82.91)	0.84
			Cumulative energy in PN + EN (kcal/kg/d)	99.25 ± 11.45	98.29 ± 12.70	0.80
			Cumulative amino acid in PN + EN (g/kg/d)	-	-	-

*TP transition phase, EUGR extrauterine growth restriction, AA amino acids, PN parenteral nutrition, EN enteral nutrition*

### Relationships between TP nutrient intake and growth outcomes

Although some significant differences and associations with TP growth were reported between studies, the findings were inconsistent. However, relative to the decrease in energy intake, the finding of decreased protein intake during the TP was consistent across studies. Only two studies reported the difference in cumulative nutrient intake (PN + EN) in the TP between individuals with and without EUGR/ inadequate weight growth velocity [13, 19]. A study found that protein intakes gradually decreased, while energy intake during the TP were comparable to baseline parenteral provision [13]. Another study reported that preterm infants with inadequate weight growth velocity had lower cumulative energy and protein (PN + EN) in the TP [19].

Two studies reported the difference in nutrient intakes (PN or EN) during the early and late stages of the TP between individuals with and without EUGR/ inadequate weight growth velocity. One of the studies reported that in the early TP, the enteral energy and amino acids were lower for preterm infants with EUGR. In the late TP, there was no significant difference [20], as shown in Table 3. The other study reported that in the early TP, the enteral protein intake was lower for preterm infants with inadequate weight growth velocity [19], as shown in Table 3.

One study found that, after controlling for gestational age and birth weight Z-score, a low Cit concentration during the TP could predict EUGR using multivariate linear regression analysis [20]. Further regression analysis was not performed in the other studies to determine the association between energy or protein intake in the TP and EUGR/inadequate weight growth velocity after controlling for confounding variables such as birth weight Z-score.

One study reported that poor growth (weight at the end of the TP < 10th percentile for post-menstrual age) in the TP rather than the PN-only phase was predictive of EUGR when adjusted for gestational age, birth weight and severity of illness [13].

## Discussion

All studies divided the nutritional phases into the PN-only phase, TP, and full EN phase, highlighting that more attention should be paid to the TP. All studies evaluated TP nutrient intake (energy, protein, or amino acid metabolism). Although findings across studies were heterogeneous (whether there is a significant difference in cumulative energy intake), in general, cumulative protein (PN + EN) intake in the TP was significantly lower in very preterm infants with and without EUGR / inadequate weight growth velocity, especially the enteral protein intake in the early TP [19, 20]. Unfortunately, except for one study that found that a low Cit concentration can predict EUGR, no other study has used regression analysis to determine the association between energy or protein intake in the TP and EUGR/ inadequate weight growth velocity with adjusting for confounding variables such as birth weight Z-score. However, poor growth (weight at the end of the TP < 10th percentile for post-menstrual age) in the TP was predictive of EUGR when adjusted for gestational age, birth weight, and severity of illness, compared to the poor growth in the PN-only phase [13].

Several previous studies investigated the relationship between nutrition and postnatal growth of preterm infants during hospitalization [24, 28–32]. Studies on nutritional practice are primarily based on early postnatal nutrition. Higher protein and energy intake in the first few weeks after birth results in better weight gain and brain development [31, 32]. The current review found that preterm infants often have interruptions of PN during the first two or four weeks after birth. Similarly, another TP study found that using a nutrition phase approach to evaluate nutritional support could reveal the timing and magnitude of nutritional deficits [15]. Nutritional deficiency interventions are categorized according to age-driven applications based only on the PN or EN phases without considering the key TP. Furthermore, it does not reveal nutritional deficiencies but approaches or exceeds the amount recommended by the guide. In contrast, another study involving the TP indicates a considerable lack of macronutrients and energy in the TP, negatively affecting postnatal growth [8, 15]. Instead of the PN and EN phases, the TP is considered to predict EUGR at discharge [13]. Miller et al. [14] proved that poor growth in the TP is related to both protein and energy deficiency. Strengthening nutritional management in the TP may improve the extrauterine growth of preterm infants.

The systematic review found the heterogeneity of growth indicators in TP. The two included studies used EUGR (weight < 10th percentile) [13, 20], and the other study used growth velocity, thus defining postnatal growth failure (growth velocity less than 15 g/kg/d) [19]. However, because the reduction in growth velocity was not the same as attaining a certain percentile, the definition of postnatal growth failure in preterm infants remained ambiguous [33]. Furthermore, there was considerable disagreement about which classification was used to define the EUGR of preterm infants and whether growth velocity less than 15 g/kg/d was used to define growth failure [33–37]. The current challenge in evaluating the growth of preterm infants in the TP is to select a growth indicator that can not only reasonably evaluate the growth of preterm infants but is also suitable for a wide range of preterm infants. Landau-Crangle et al. [38] proposed the necessity of monitoring the individualized growth trajectory of preterm infants after birth. They believed that calculating the growth velocity could provide an evidence-based starting point for growth monitoring and the nutritional evaluation of preterm infants. However, in a comparative study that selects the change of weight z-score or a Patel exponential model to evaluate the growth of preterm infants, it is considered that the change in weight z-score takes into account gestational age and sex, making it suitable for analyzing a population of preterm infants with a wide range of gestational ages [39]. To reduce the risk of bias due to confounding factors, the two included studies excluded small gestational-age infants from the very preterm infants less than 32 weeks [13, 20]. Small for gestational age or suitable for gestational age infants might have different postnatal growth outcomes [40]. To facilitate the establishment of nutritional guidelines in the TP, clinicians must choose a reasonable evaluation indicator in combination with individual characteristics to objectively assess the nutritional status of preterm infants [41].

The TP is the reduction of PN and the transition to EN. For the three studies included in this systematic review, the starting point of the TP was relatively uniform, which is the reduction of parenteral nutrition or the volume of enteral feeding up to 30 ml/kg/d, both after minimal enteral feeding; however, the endpoint of the TP is still controversial. Brennan et al. [15, 16] and Alur et al. [18] considered that the endpoint of the TP was that the volume of enteral feeding reached 120 ml/kg/d and determined that the TP was the key period of massive nutrient deficiency. According to Miller et al. [13] and Liotto et al. [19], the quantitative definition of TP is that enteral feeding volume gradually increases to adequate enteral feeding (160 ml/kg/d) after minimal enteral feeding. However, in practice, when the actual enteral feeding volume of preterm infants reaches 100–120 ml/kg/d, PN can be considered to be stopped because, at this time, enteral feeding provides sufficient protein intake, that is, 3.5 g/kg/d [4, 16, 22]. Therefore, the TP can be defined quantitatively as 30 ml/kg/d ≤ enteral feeding volume ≤ 120 ml/kg/d.

Brennan et al. [15, 16] suggested that the TP is divided into the early period, dominated by PN (enteral feeding volume < 80 ml/kg/d, main parenteral nutrition intakes), and the late period dominated by EN (enteral feeding volume ≥ 80 ml/kg/d, main enteral nutrition intakes). During early TP, a lower Cit concentration was positively correlated with the compromised protein and energy deficits in EN [20]. To further investigate the nutrition and growth of preterm infants, the TP is divided into early and late periods.

The three phases ( PN, transition from PN to EN, and full EN) are not gradual for preterm infants. These three phases may be experienced repeatedly by some preterm infants due to intolerance to food or diseases. It is necessary to adjust TP for preterm infants according to local conditions.

To the author’s knowledge, this is the first comprehensive assessment of nutrition in the TP and growth of preterm infants. Generally, preterm infants with EUGR or inadequate weight growth velocity consumed less protein in the early postnatal period. Therefore, under the background that there are currently no nutritional management guidelines for TP, strengthening the EN intake in the early TP [17], increasing human milk fortifier when the volume of human milk reaches 50–80 ml/kg/d　[42], and standardizing the composition of PN in the TP [16, 19] could achieve optimal nutrient intake. It has also been reported that early progressive enteral feeding with human milk is well tolerated by preterm infants, which may improve growth in the hospital [43]. Additionally, the duration of TP varied among the studies included in this systematic review. According to another study comparing the nutritional status of preterm infants in Chinese and American hospitals, Chinese preterm infants had significantly longer PNs than American preterm infants [44]. However, there were no comparative data on the duration of the TP. There is a need to conduct relevant research on the TP of preterm infants, identify nutrition gaps in different regions, and investigate whether preterm infants' TP duration affects their growth.

There are some limitations to our study. Firstly, the studies included in this systematic review had small sample sizes and were all single-center, observational studies. Further large-scale, prospective, and intervention studies are needed to guide the nutritional management of preterm infants during hospitalization. Second, a meta-analysis of the data was not feasible due to the heterogeneity between studies, the different nutrition strategies, and the inconsistent definitions of the TP. Therefore, this review only provides a narrative summary of the results. The third limitation of this systematic review is that it only conducted bivariate analyses between nutrition and PGF in the included studies. The current findings do not fully consider the presence of infants with SGA, the development of comorbidity, or the type of enteral feeding. More multivariate analysis of transitional nutrition and growth outcome is needed to determine the independent risk factors of growth failure. Finally, in the studies involving nutritional intake in the late TP included in this review [19, 20], it is still controversial whether the energy and protein intake in the late TP is related to PGF. There are no specific indicators to evaluate protein-energy status in the current research, such as BUN, prealbumin, and albumin. Therefore, it is necessary to conduct extensive sample studies to clarify the relationship between nutrition intake in the late TP and growth outcome. Considering these limitations, the results should be interpreted accordingly.

## Conclusion

Nutrient intake differences were observed in the TP, including energy, protein, and amino acid metabolic patterns. Nevertheless, considerable heterogeneity was due to differences in growth indicators assessed and definitions of TP. Furthermore, due to the small number of studies retrieved by the review, it is difficult to determine whether a lack of energy and protein intake in the TP can predict poor growth compared to nutrient intake in the PN-only and EN phases. This is the first systematic review to integrate several definitions of the TP from PN to EN. The conclusion is that the definition (30 ml/kg/d enteral feeding volume 120 ml/kg/d) is more reasonable. Generally, preterm infants with extrauterine growth restriction or inadequate weight growth velocity showed lower enteral protein intake in the early TP. Furthermore, this review emphasizes the need for additional high-quality study designs that include rigorous growth assessment methodology to elucidate potential associations between nutrition during the TP and the growth of very preterm infants.

## Data Availability

The datasets used and/or analyzed during the current study are available from the corresponding author on reasonable request.

## Abbreviations

TP: Transition phase
PGF: Postnatal growth failure
EUGR: Extrauterine growth restriction
AA: Amino acids
PN: Parenteral nutrition
EN: Enteral nutrition
SGA: Small for gestational age
